# A Longitudinal Study of the Reliability of Acupuncture Deqi Sensations in Knee Osteoarthritis

**DOI:** 10.1155/2013/204259

**Published:** 2013-07-01

**Authors:** Rosa B. Spaeth, Stephanie Camhi, Javeria A. Hashmi, Mark Vangel, Ajay D. Wasan, Robert R. Edwards, Randy L. Gollub, Jian Kong

**Affiliations:** ^1^Department of Psychiatry, Massachusetts General Hospital, Charlestown, MA 02129, USA; ^2^Department of Psychology, Endicott College, Beverly, MA 01915, USA; ^3^MGH/MIT/HMS Athinoula A. Martinos Center for Biomedical Imaging, Charlestown, MA 02129, USA; ^4^Department of Radiology, Massachusetts General Hospital, Charlestown, MA 02129, USA; ^5^Departments of Anesthesiology, Perioperative and Pain Medicine and Psychiatry, Brigham and Women's Hospital and Harvard Medical School, Boston, MA 02115, USA

## Abstract

Deqi is one of the core concepts in acupuncture theory and encompasses a range of sensations. In this study, we used the MGH Acupuncture Sensation Scale (MASS) to measure and assess the reliability of the sensations evoked by acupuncture needle stimulation in a longitudinal clinical trial on knee osteoarthritis (OA) patients. The Knee injury and Osteoarthritis Outcome Score (KOOS) was used as the clinical outcome. Thirty OA patients were randomized into one of three groups (high dose, low dose, and sham acupuncture) for 4 weeks. We found that, compared with sham acupuncture, real acupuncture (combining high and low doses) produced significant improvement in knee pain (*P* = .025) and function in sport (*P* = .049). Intraclass correlation analysis showed that patients reliably rated 11 of the 12 acupuncture sensations listed on the MASS and that heaviness was rated most consistently. Overall perceived sensation (MASS Index) (*P* = .014), ratings of soreness (*P* = .002), and aching (*P* = .002) differed significantly across acupuncture groups. Compared to sham acupuncture, real acupuncture reliably evoked stronger deqi sensations and led to better clinical outcomes when measured in a chronic pain population. Our findings highlight the MASS as a useful tool for measuring deqi in acupuncture research.

## 1. Introduction

Deqi (obtaining qi) is a core concept in traditional Chinese acupuncture theory [1, 2] that describes the physiological link between the stimulation of acupuncture needles and the energy meridians running through the body [2–5]. The term deqi encompasses numerous sensations (e.g., soreness, heaviness), the complete range of which is debated [6–8]. 

Traditional ancient acupuncturists believed that deqi was comprised of sensations and/or experiences of both the patient receiving the treatment and the acupuncturist administering the treatment [7–9]. Modern acupuncturists and researchers, however, have emphasized the patient's sensations rather than the acupuncturist's experience during needling [10–13]. One challenge in investigating these acupuncture sensations is that perception of deqi is subjective and the specific sensations associated with deqi may vary significantly both between and within individuals, which calls for the development of a systematic measurement of deqi sensation. To overcome this barrier, in recent years, investigators have started to use different scales to measure deqi sensation [2, 10–16] and have investigated the association between deqi sensation and therapeutic effects [13, 17, 18].

It is generally believed that deqi sensation is crucial for effective acupuncture treatment, a belief rooted in traditional Chinese acupunture theory [2]; however, the link between these sensations and improvements in clinical outcomes remains unclear [19, 20]. Previous studies investigating the relationship between deqi sensations and clinical outcomes are contradictory [21–24]. It is important to note that most studies use deqi as a general construct [25] and that none of these studies explored the association between clinical outcomes and specific, quantified sensations [21–24]; rather, these studies investigated the difference between traditional Chinese acupuncture (with deqi) and sham acupuncture (with no or minimal deqi sensation). In a previous study in healthy subjects, we developed an acupuncture sensation scale [13] to measure the sensations evoked by electroacupuncture, manual acupuncture, and sham acupuncture. This scale has subsequently been revised, renamed, and used in other acupuncture research studies including the present study [2, 26]. In our previous study, we found that numbness and soreness were significantly associated with analgesia to experimental heat pain [13]. Nevertheless, few studies have systematically measured and characterized deqi sensations in a patient population longitudinally and explored the association between quantified deqi sensations and clinical outcomes.

In the present study, we longitudinally investigated acupuncture treatment-evoked deqi sensations in a chronic pain population using the Massachusetts General Hospital (MGH) Acupuncture Sensation Scale (MASS) and explored the association between deqi sensations and changes in clinical outcomes related to knee pain. More specifically, knee osteoarthritis (OA) patients were randomized into one of three treatment groups: high-dose acupuncture treatment (application of six acupuncture points), low-dose acupuncture treatment (application of 2 acupuncture points), and sham acupuncture (Streitberger placebo acupuncture needles on 6 nonacupoints). We employed a tapered longitudinal treatment design [21], such that each patient received 6 acupuncture treatments over the course of 4 weeks (2 treatments per week for the first 2 weeks and one treatment per week for the last 2 weeks). Deqi sensations were measured using the MASS twice during each treatment. And after the six-session acupuncture treatment period, the Knee injury and Osteoarthritis Outcome Score (KOOS) was also administered to investigate changes in knee pain and function following treatment with either real or sham acupuncture.

## 2. Materials and Methods

### 2.1. Subjects

The Institutional Review Board at the Massachusetts General Hospital approved all study procedures. All subjects provided written informed consent at the beginning of the study and were debriefed at the end of the study. 

### 2.2. Patient Recruitment and Inclusion Criteria

Acupuncture naïve patients aged 40–70 with a diagnosis of chronic painful osteoarthritis (OA) in the right and/or left knee were recruited for this study, as previous studies have indicated that acupuncture is an effective treatment for patients with chronic knee pain [21–23]. Investigators excluded acupuncture-experienced subjects to minimize the possibility of subjects distinguishing sham from real acupuncture, serving to assist in blinding the subjects to their assigned treatment group. Patients were recruited from the Massachusetts General Hospital (MGH) and Brigham and Women's Hospital (BWH). 

Subjects were included if they met the Kellgren-Lawrence scale for radiographically grading knee OA [27–29] as grade 2 or 3. Those with severe knee OA were excluded. Other specific inclusion and exclusion criteria were designed to allow for the retention of a relatively homogenous clinical population; subjects were excluded for any interventional procedure for knee pain within 6 months prior to enrolling in the study, intent to undergo surgery during the time of involvement in the study, knee pain due to other causes such as inflammation or malignancy, diagnosis of rheumatoid arthritis or other pain disorders that may refer pain to the leg, medications or disorders that would put patients at heightened potential for adverse outcome, and presence of MRI contraindications (e.g., cardiac pacemaker, metal implants, claustrophobia, and pregnancy). All OA patients had an endogenous knee pain intensity rating (average in the last week) of >2 on a 0 to 10 scale at the first visit. 

### 2.3. Experimental Design

**Figure 1:**
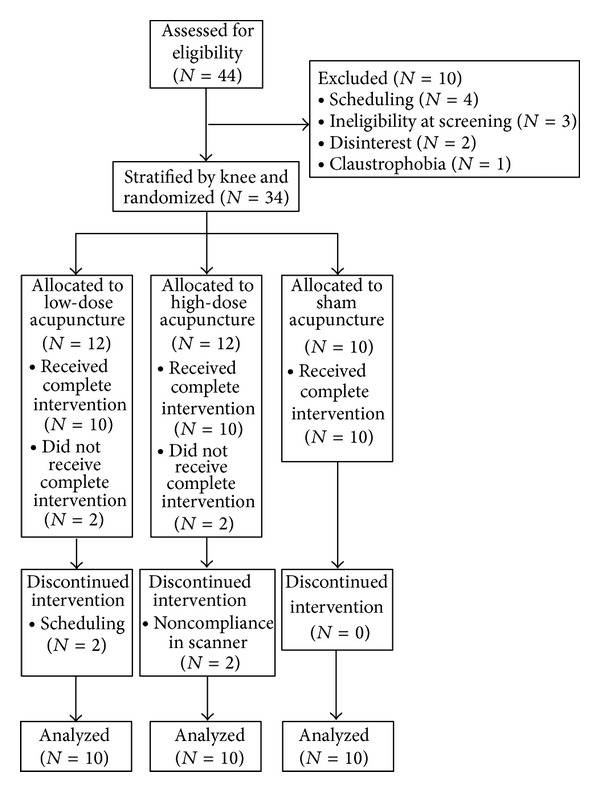
Consort diagram indicating the number of patients enrolled, dropped, and completed, by group.

To maintain consistency within our sample of patients who had both unilateral and bilateral knee pains, we only treated one knee for each subject. For those subjects with bilateral knee pain, the knee with the highest pain ratings was treated. Subjects were stratified by knee and randomized into one of the three groups: high-dose real acupuncture (6 acupoints), low dose real acupuncture (2 acupoints), and high-dose sham acupuncture (6 nonacupoints with Streitberger placebo needles) (see Figure 1).

### 2.4. Blinding

At the time of consent, all patients were informed that they would receive one of three modes of acupuncture treatment and that there was an equal chance of receiving each mode of treatment. Using specially designed placebo needles (described below) and acupuncture-naïve subjects, we were able to keep all subjects blinded to acupuncture mode (real versus sham acupuncture). Subjects were not told how many needles would be used in the high- versus low-dose acupuncture groups. All clinical outcomes detailed below were measured by research staff, also blinded to treatment condition; thus, the study was single blinded (patients and research staff were blinded; acupuncturist was not blinded).

After an initial screening session, each subject engaged in a total of 6 acupuncture-treatment sessions, completing the MASS form twice within each session. Treatments 1, 3, and 6 occurred approximately 15 minutes into a scan session in which the patient was lying in a 3 Tesla Tim Trio magnetic resonance imaging scanner (Siemens, Erlangen, Germany) while functional imaging data was acquired. The remaining treatments were administered in a behavioral testing room with patients reclined in a chair. All acupuncture treatments were completed within four weeks.

### 2.5. Acupuncture Administration

**Figure 2:**
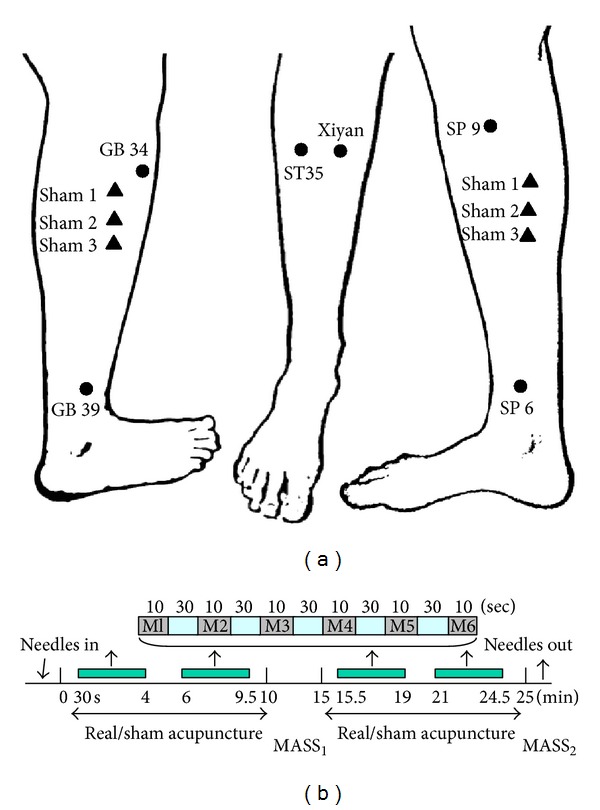
Standardized acupuncture protocol. (a) Real and sham acupuncture points. Low-dose real acupuncture was applied on ST35 and Xiyan (extra point). High-dose acupuncture group was applied to four additional points: GB34, SP9, GB39, and SP6. Six sham acupuncture points were used for the sham acupuncture group. (b) Acupuncture stimulation paradigm for both real and placebo acupunctures, indicating the timeline of intermittent needle stimulation during each acupuncture treatment. Six 10-second periods of manual needle rotation (M) were separated by 30 seconds of rest. The manual stimulation series (M1–6) was repeated a total of 4 times, twice prior to administering the first MASS, and an additional 2 times prior to the second MASS.

High- and low-dose acupuncture groups differed only in the number of acupoints stimulated. In the high-dose group, 6 needles were inserted at 6 acupoints (see Figure 2(a)), and each point was stimulated 4 times. In the low-dose group, 2 needles were inserted, and each point was stimulated a total of 12 times. The total length of the treatment remained constant across all treatment groups. All other treatment parameters, as described below, were held constant (Figure 2(b)).

Each acupuncture treatment session for subjects in both the real and the sham acupuncture groups was about 25 minutes in duration and was carried out by the same licensed acupuncturist. For all treatments, the acupuncturist located the acupoints on the leg, disinfected each point with isopropyl alcohol, and then placed a small plastic ring over the point, securing the ring with a thin strip of sterile plastic tape. This ensured patient blindness to the actual site of needle insertion and thus blindness to whether the treatment was real or sham. For all patients, a predetermined number of acupoints (either 2 or 6) were stimulated in a predetermined order for a total of 24 stimulations (Figure 2(b)). 

#### 2.5.1. Real Acupuncture Treatments

For the low-dose acupuncture group, real acupuncture was applied to ST35 and Xiyan (extra point) (see Figure 2(a)). These two acupuncture points are well documented for treating knee pain according to traditional Chinese acupuncture [9, 30]. Both points, located near the knee, have been used in previous clinical trials of OA patients [21–23]. 

The high-dose acupuncture group received treatment at four additional points including GB34, SP9, GB39, and SP6 (see Figure 2(a)). 

For consistency, leg position, acupoint location, and needling parameters (1-2 cm depth, approximately 120 rotations per minute, 90° insertion angle, and moderate deqi sensations on a 0–10 scale) were kept constant across groups. Needles were rotated at each point for 10 seconds with 30-second breaks between each point (see Figure 2(b)). All points (either 2 or 6 acupoints) were stimulated one point at a time and were stimulated in a predetermined order. In the high-dose group, needles were manipulated in the order of GB34, SP9, ST35, Xiyan (extra point), GB39, and SP6. The specific starting acupoint was randomized across patients, but within patients, the starting point was held constant across sessions. 

#### 2.5.2. Placebo Acupuncture Procedures

Placebo acupuncture was applied at six nonacupoints using Streitberger placebo needles specially designed for subject blinding [13, 31–35] using a paradigm identical to the real acupuncture treatment. These sham needles are visually indistinguishable but differ from regular needles by virtue of their blunt, retractable tip. Instead of penetrating the skin, the point of the Streitberger needle retracts up into the shaft when the acupuncturist presses it against the skin. This sham device has been validated by studies showing that subjects cannot distinguish between real and sham needling [13, 31, 32].

Six sham points were used during placebo acupuncture: sham point 1 was located 1.5 cun posterior and inferior to GB34, sham points 2-3 were located 1.5 cun and 3 cun inferior to sham point 1, sham point 4 was located 1 cun posterior to the midpoint of K9 and K10, and sham points 5-6 were located 1.5 cun inferior and superior to the sham point 4 separately (see Figure 2(a)). All sham points were located on the lower leg where no meridians pass through. Placebo treatment administration was similar to high-dose acupuncture administration with regard to the number of acupoints.

### 2.6. Clinical Outcomes

#### 2.6.1. Knee Injury and Osteoarthritis Outcome Score (KOOS)

The Knee injury and Osteoarthritis Outcome Score (KOOS) [36] was used to measure clinical outcomes. The KOOS is comprised of 5 subscales, each of which produces an outcome score. These subscales include pain, other symptoms, function in daily living (ADL), function in sport and recreation, and knee-related quality of life (QOL). Based on previous studies, subscale scores of the KOOS related to pain, function in daily living, and function in sport and recreation were selected as the primary outcome of the present study [21]. Other subscores were used for exploratory analyses. Trained research assistants, blinded to treatment mode, administered the KOOS to all patients at baseline (within one week of the first treatment) and at the final (sixth) treatment. For each subscale, a normalized score was calculated, where 0 indicated the most extreme symptoms/pain and 100 indicated no symptoms/pain [36]. 

#### 2.6.2. Massachusetts General Hospital Acupuncture Sensation Scale (MASS)

The Massachusetts General Hospital (MGH) Acupuncture Sensation Scale (MASS) [2] is the revised version of the Subjective Acupuncture Sensation Scale that has been used in previous studies in healthy subjects [13]. This scale includes 12 descriptors (soreness, aching, deep pressure, heaviness, fullness/distension, tingling, numbness, sharp pain, dull pain, warmth, cold, and throbbing) that are considered to be associated with acupuncture treatment and one supplementary field for subjects to describe the acupuncture sensation in their own words [2]. Subjects were asked to rate the intensity of each sensation on a scale from 0 to 10, where 0 is none and 10 is unbearable. This scale was created through a collaboration of acupuncture researchers at the MGH Martinos Center and has been used by acupuncture researchers since 2007 [26, 37]. The MASS has subsequently been translated and validated in Chinese [38]. 

All subjects were asked to rate their acupuncture sensations using the MASS twice during each treatment. Subjects were asked to report the average sensation across all of the needles used for their treatment (either 2 or 6, depending on the group). Prior to the first treatment, the acupuncturist gave all subjects a brief description of deqi, as subjects were acupuncture naïve upon enrollment. Each subject was told the following: “The MGH Acupuncture Sensation Scale lists 12 of the sensations commonly reported by people who receive acupuncture. Different patients experience different sensations, and you might not experience all of the sensations. If you feel a sensation that is not listed here, you may write in the sensation you feel and indicate how intensely you felt that sensation.” The MASS was used to measure average sensations *during* needle stimulation across each 10-minute treatment period. After the first block of intermittent acupuncture stimulation (see Figure 2(b)), subjects were asked to indicate the extent to which each of the 12 descriptors described their subjective acupuncture experiences. Subjects were asked to repeat this assessment again after the second 10-minute block of intermittent stimulation.

The MASS index is a measure that describes the overall magnitude of deqi sensation experienced during treatment. Using previously described methods [2], the index was calculated by ranking all of the sensations by self-reported intensity ratings (0–10) and then assigning a weight to each sensation based on rank. 

### 2.7. Data Analysis

Statistical analyses were performed using SPSS 18.0 Software (SPSS Inc., Chicago, IL, USA). Variance in baseline characteristics, intensity ratings of each individual sensation, the overall sum of all sensations, and the MASS index across treatment modalities were analyzed using a one-way ANOVA and post hoc *t*-tests (*P* < .05) and were corrected for multiple comparisons. All confidence intervals (CIs) are reported at the 95% confidence level. 

The MASS index, a weighted average of the intensity of sensations elicited, was calculated using previously published methods [2]. In brief, for each administration of the MASS (12 per subject), the deqi sensation descriptors (soreness, aching, etc.) were ordered from the highest to the lowest subjective intensity rating. As previously suggested, ratings of sharp pain were excluded from the MASS index calculation, as sharp pain is not always considered a deqi sensation. Using exponential smoothing, a weighted average (MASS index) was calculated.

The internal consistency reliability of the MASS scale was computed and results are presented as Cronbach's alpha. Measures of the test-retest reliability of each individual sensation, the overall sum of all sensations, and the MASS index were computed using intraclass correlation coefficients. To compare how frequently each sensation was rated ≥1 on a scale from 0 to 10 across the 3 groups, a chi-square test for independence was conducted for each sensation. For each chi-square test, we compared the number of people who reported that sensation at least once throughout the 6 treatments across the three groups.

Factor analysis was performed using the principal component extraction method to segment acupuncture sensations (MASS) into meaningful clusters. Component extraction was based on eigenvalues greater than 1.0 with no specifications for a fixed number of factors to extract. A Varimax rotated solution with 25 maximum iterations for convergence was analyzed. Factors were loaded with a cut-off value of 0.4 (representing 16% variance). Pearson's correlations were applied to examine the potential relationship between osteoarthritis treatment outcomes (KOOS) and the perceived intensity of select sensations identified by the principal component analysis (PCA). 

## 3. Results

Forty-four (19 females) acupuncture naïve patients aged 43–70 with a diagnosis of chronic painful osteoarthritis in the right and/or left knee enrolled in the study. Of the 44 patients who enrolled, 30 (13 females) completed all study procedures. Ten of the 14 patients who did not complete all study procedures dropped out prior to the first treatment due to ineligibility at screening (3), scheduling conflicts (4), claustrophobia (1), and lack of interest (2); the remaining 4 patients who underwent at least one acupuncture treatment session dropped out for the following reasons: scheduling conflicts (2, low-dose group) and inability to adhere to study requirements in scanner (2, high-dose group) (see Figure 1).

### 3.1. Clinical Outcomes

Of the 30 patients who completed all study procedures, 20 were treated on their right knee, and 10 were treated on their left knee. Baseline characteristics are detailed in Table 1. One subject in the low-dose acupuncture group did not complete the KOOS subscale for function in sport; thus, for all analyses including the KOOS function in sport variable only complete datasets (*N* = 29) were used.

**Figure 3:**
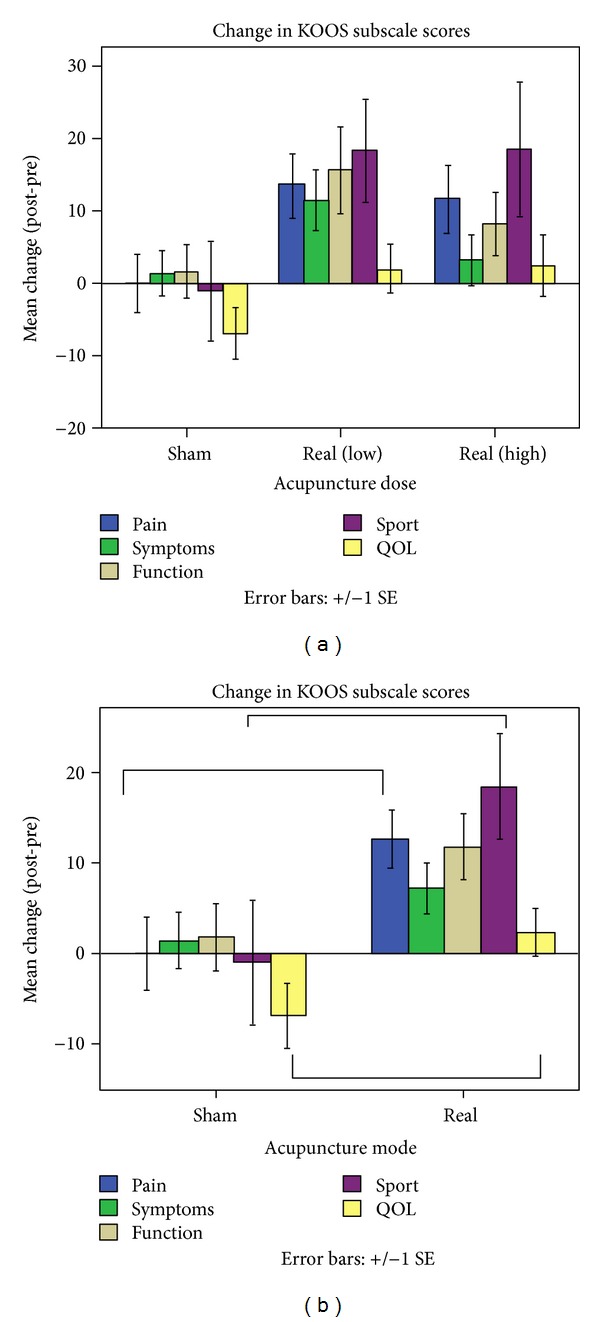
Changes in KOOS subscale scores from baseline to endpoint. Improvement in each of the 5 domains is indicated by a positive value. (a) The interaction between group (high versus low versus sham) and time (baseline versus endpoint) showed a trend for the KOOS pain subscale (*F*(2,27) = 2.709, *P* = .085) but not for either function in daily living (*F*(2,27) = 2.178, *P* = .133) or function in sport (*F*(2,26) = 2.047, *P* = .149). (b) The interaction between group (real versus sham) and time (baseline versus endpoint) was significant for the KOOS subscale scores for pain (*F*(1,28) = 5.596, *P* = .025), function in sport (*F*(1,27) = 4.252, *P* = .049), and quality of life (QOL) (*F*(1,28) = 4.682, *P* = .039).

Repeated measurements analysis of pre- and post-treatment knee pain across three groups revealed a significant effect of time (baseline versus endpoint) on the KOOS subscales for pain (*F*(1,28) = 9.661, *P* = .004, and 95% CI [2.75, 13.34]), function in daily living (ADL) (*F*(1,28) = 8.310, *P* = .007, and 95% CI [2.61, 13.92]), and function in sport (*F*(1,28) = 6.145, *P* = .0019, and 95% CI [2.04, 21.41]). A trend was observed for the interaction between group and time on the KOOS pain subscale score (*F*(2,27) = 2.709, *P* = .085) but not for either function in daily living (*F*(2,27) = 2.178, *P* = .133) or function in sport (*F*(2,26) = 2.047, *P* = .149) (see Figure 3(a)). Post hoc tests indicated no significant differences between the high- and low-dose real acupuncture groups for pain (*P* = .612), function in daily life (*P* = 1.0), and function in sport (*P* = 1.0).

To increase power in our analysis, we combined the two real acupuncture groups (high and low dose) to compare real acupuncture (*N* = 20) to sham acupuncture (*N* = 10). The results indicated a significant interaction between acupuncture mode (real versus sham) and time (baseline versus endpoint) on our primary outcomes: the KOOS subscale scores for pain (*F*(1,28) = 5.596, *P* = .025) and function in sport (*F*(1,27) = 4.252, *P* = .049). In addition, we found that our secondary outcome (KOOS subscale score for quality of life (QOL)) showed significant improvement in the real acupuncture group after treatment compared with the sham group (*F*(1,28) = 4.682, *P* = .039) (see Figure 3(b)).

### 3.2. Acupuncture Deqi Sensations

In this study, subjects reported deqi sensations at 2 different time points in each treatment session: after the first 10-minute acupuncture stimulation period and again after the second 10-minute acupuncture stimulation period (see Figure 2(b)). In total, 30 subjects completed 6 treatment sessions, and all but one subject completed the MASS twice per treatment. For one subject, the second deqi assessment was missing from 3 treatment sessions. For the internal consistency reliability analysis of the MASS, only complete data sets were used. For all other analyses, the first and second administrations of the MASS in each treatment were averaged for each sensation.

#### 3.2.1. Internal Consistency Reliability of the MGH Acupuncture Sensation Scale (MASS)

The Cronbach's alpha reliability of the 12 items in the MASS was calculated for each administration of the MASS (twice per treatment session) and ranged from 0.856 to 0.948 (see Table 2 for complete list of Cronbach's alphas).

#### 3.2.2. Test-Retest Reliability of Deqi Sensation across Different Treatment Modes

Intraclass correlation analysis showed that patients rated soreness, aching, deep pressure, heaviness, fullness/distension, tingling, numbness, sharp pain, dull pain, warmth, and throbbing reliably across all 6 sessions (ICC ranged from .928 to .768). In short, 11 of the 12 sensations on the MASS were rated reliably with the exception of cold (ICC = .078, *P* = .37), and heaviness was rated most reliably across all sessions (ICC = .928, *P* < .001). 

Further analysis of each treatment group indicated that, in the high-dose acupuncture group, heaviness (ICC = .88, *P* < .001) was rated the most reliably. In the low-dose acupuncture group, deep pressure (ICC = .943, *P* < .001) and fullness/distention (ICC = .943, *P* < .001) were rated the most reliably. In the sham acupuncture treatment group, numbness was rated most reliably (ICC = .932, *P* < .001). None of the treatment groups reliably rated the cold sensation. Both the sum score and the MASS index were highly reliable across all subjects and within each group (see Table 3 for complete list of all intraclass correlation coefficients). 

#### 3.2.3. Intensity of Sensations

Across all 30 patients, the sensations that were rated with the highest intensity in response to treatment included soreness, dull pain, and sharp pain. Those rated at the lowest intensity included cold and warmth. Descriptive statistics for each sensation are listed in Table 4.

After Bonferroni corrections for multiple comparisons (*P* < .0038), intensity ratings of soreness (*F*(2,27) = 7.74, *P* = .002) and aching (*F*(2,27) = 7.55, *P* = .002) differed significantly across treatment groups (high versus low versus sham). Post hoc comparisons of soreness and aching demonstrated that there was no significant difference between the high- and low-dose real acupuncture groups and that those subjects in the sham group reported significantly less soreness and aching as compared to the high-dose (*P* = .01 and *P* = .05, resp.) and low-dose (*P* = .003 and *P* = .002, resp.) acupuncture treatment groups. Reported intensity ratings for each individual sensation are depicted in Table 4. 

To further elucidate the differences between real and sham acupuncture, data from the low- and high-dose acupuncture groups were pooled to increase power. After correction for multiple comparisons (*P* < .0038), sensations of soreness (*P* < .001), aching (*P* < .001), and throbbing (*P* = .003) were rated significantly more intensely in the real acupuncture group compared to the sham acupuncture group. 

The average total MASS score (sum of the intensities of each sensation) differed significantly across the acupuncture treatment groups (high versus low versus sham) (*F*(2,27) = 4.21, *P* = .026). Similarly, the MASS index, or overall perceived sensation of acupuncture, differed significantly across the acupuncture treatment groups (*F*(2,27) = 5.03, *P* = .014). Those who received sham acupuncture had a significantly lower MASS index and total MASS score than those who received either high-dose (*P* = .04 and trend *P* = .1, resp.) or low-dose (*P* = .02 and *P* = .03, resp.) acupuncture treatments. No significant difference was observed between high- and low-dose acupuncture groups for the MASS Index (*P* = .949) or the total MASS score (*P* = .617).

#### 3.2.4. Frequency of Sensations

A chi-square test for independence indicated that there was a significant effect of acupuncture dose (high versus low versus sham) on the number of individuals reporting soreness (*χ* ^2^ (2, *N* = 30) = 6.24, *P* = .044) and that there was a trend for aching (*χ* ^2^ (2, *N* = 30) = 5.96, *P* = .051) and fullness/distension (*χ* ^2^ (2, *N* = 30) = 5.83, *P* = .054). Further comparisons between acupuncture modes (real versus sham) indicated that there was a significant effect of acupuncture mode on frequency of reporting soreness, aching, and fullness/distension (*P* < .05). The total number of individuals reporting each sensation is listed in Table 5. 

A one-way ANOVA comparing the total number of sensations reported by each subject across treatment groups (high versus low versus sham) revealed a trend in the effect of acupuncture dose (*F*(2,27) = 2.68, *P* = .087). Further comparison of the real and sham acupuncture treatment groups using an independent sample *t*-test (equal variances not assumed according to Levene's test for equality of variance) showed that those who received real acupuncture reported significantly more sensations during treatment (*t*(25.37) = −2.65, *P* = .014) compared to the sham acupuncture group. Subjects who received real acupuncture reported an average of 5.79 ± 3.27 (mean ± SD) sensations during each treatment, while those who received sham reported experiencing 3.12 ± 2.19 sensations. 

#### 3.2.5. Principal Component Analysis

To further investigate the clustering of deqi sensations, principal components analysis (PCA) with Varimax rotation of components and Kaiser normalization was applied to the acupuncture sensations for all subjects. Two components with eigenvalues greater than 1.0 were identified, accounting in total for 77.4% of the variance. In this analysis, we used a factor loading cutoff of 0.4. The Kaiser-Meyer-Olkin (KMO) measure of sampling adequacy was .824, and Bartlett's test of sphericity was significant (*χ* ^2^ (66) = 385.65, *P* < .001) indicating that the data were suitable for factor analysis. While some variables loaded on a single factor, other variables loaded on both factors, providing evidence of the complex nature of some of the sensations (see Table 6). The variables loading onto a single factor can be characterized either by deep pressure sensations (heaviness, fullness/distension, dull pain, and cold) or sensations related to “spreading sensations” (tingling, throbbing). 

Due to the similarity between high- and low-dose acupuncture with regard to intensity of sensations, we grouped the subjects receiving real acupuncture (both high and low dose) and added acupuncture mode (real or sham acupuncture) as a variable in our model to determine whether any of the sensations elicited were related to a single mode of acupuncture. The results of the PCA using acupuncture mode as an additional variable identified three components with eigenvalues greater than 1.0, accounting for a total of 82% of variance (see Table 7). The KMO measure of sampling adequacy was .824, and Bartlett's test of sphericity was significant (*χ* ^2^ (78) = 396.50, *P* < .001), again indicating that, after including acupuncture mode, the data were still suitable for factor analysis. Again, variables related to localized deep pressure sensations loaded onto the first factor, and variables related to spreading sensations loaded onto the second factor. Two complex variables (aching and soreness) loaded onto the third factor with acupuncture mode, providing further evidence that aching and soreness are associated with acupuncture mode (real versus sham). 

#### 3.2.6. Relation to Clinical Outcomes

To explore the relationship between acupuncture sensations and clinical outcomes, we performed Pearson's correlation analyses on the MASS index and KOOS subscales across all three groups. Results showed that there were no significant correlations between the overall perceived intensity of sensations (MASS index) and changes (baseline versus endpoint) in any of the subscales of the KOOS. For exploratory purposes, we also performed a PCA to model whether specific sensations were related to each subscale of the KOOS. For the pain and QOL subscales, two components were identified with eigenvalues greater than 1.0 with a factor loading cutoff of 0.4, and for symptom, ADL and sport, three components were identified. For each of the KOOS subscales, the KMO measure of sampling adequacy ranged from 0.802 to 0.819, and all Bartlett's tests of sphericity were significant (*P* < .001) indicating that the additional variables were suitable for factor analysis. Changes in both pain and QOL loaded onto a factor with tingling, throbbing, and sharp pain. For changes in function in daily living and in sport, the acupuncture sensations loaded onto two different factors, and the KOOS subscale score loaded onto a third factor with a negative correlation with warmth. For changes in symptoms, the KOOS subscale score did not load onto a component with any of the sensations, indicating that it did not covary with any of the sensations (see Table 8 for the results of the PCA with each KOOS subscale included as an additional variable). 

Post hoc tests were conducted to verify the associations indicated by the PCA between the MASS sensations and each subscale of the KOOS that loaded together. This exploratory analysis revealed a significant correlation between the intensity of the throbbing sensation and endpoint QOL subscale of the KOOS controlling for baseline QOL score (*r* = .477, *P* = .009) as well as the intensity of the tingling sensation and the QOL subscale score at endpoint controlling for baseline QOL (*r* = .368, *P* = .049). No other comparisons were significant when Pearson's correlations were tested. 

### 3.3. Blinding

At the end of the study, all subjects were asked to complete a set of final questions to assess how well subject blinding was maintained throughout the study. Ninety percent (*N* = 27) of the subjects believed that the needle was inserted into the skin in every session. The 3 subjects who believed that the needle was not inserted were in the real acupuncture (low dose) group.

## 4. Discussion

In this longitudinal clinical trial, we investigated the descriptive nature of deqi in knee OA patients. We found that in real acupuncture treatment, soreness, deep pressure, dull pain, and sharp pain were the most frequently reported sensations. The intraclass correlation analysis indicated that most of the sensations on the MASS (with exception of cold) were reported reliably across different treatment sessions, implying that the deqi sensation can be reliably measured within subjects using scales such as MASS in a chronic pain population. 

In this study, the average deqi sensation was of relatively weak intensity compared to previously reported acupuncture sensations [26, 39]. For the present study, the average intensity for each sensation was between .06 and 1.89, compared to our previous studies, where average intensity of each sensation was between 0 and 4 [26] and between 0.1 and 3.7 [39]. We believe that this may be due to the age of patients in the present study (58 ± 8.3) compared to studies conducted in healthy, young subjects (29 ± 7 [26] and 26.4 ± 4.9 [39]). We speculate that one difference between these populations is that the peripheral nervous system in these older patients may not be as sensitive as other younger populations. Additionally, we note that there are differences in the specific acupoints needled and the disorder treated in this study compared to previous studies, which may also influence the intensity of the sensations reported. 

In this study, knee OA patients across all treatment groups tended to report soreness, deep pressure, tingling, dull pain, and sharp pain, among others, all of which are typical deqi sensations based on traditional Chinese medicine. Compared with real acupuncture, sham acupuncture using a placebo needle evoked very mild sensations, implying that superficial stimulation may be associated with different subjective sensations than deep tissue stimulation. This is consistent with previous studies that reported greater deqi sensations in real compared to placebo acupuncture [20]. In the present study, soreness and aching were reported as significantly more intense in the real acupuncture group compared to the sham acupuncture group. 

Our results showed that sensations were equally reliable in the low-dose real acupuncture group as they were in the sham acupuncture group, but less reliably in the high-dose real acupuncture group. This may be due to the fact that subjects were asked to report their average sensations across all of the needles, and the number of needles differed between groups. For the low-dose acupuncture group, subjects reported the average intensity of each sensation across two needles; however, for the high-dose acupuncture group, subjects reported the average intensity of each sensation across all 6 needles, which is a complex task that could add additional variability to the data. For the sham acupuncture group, subjects reported fewer sensations in total, meaning that there was less room for variability in the repeated report of sensations. 

Overall, the most reliably rated sensation was heaviness, and the least reliably rated sensation was coldness. We suspect that the reliability of the sensations may be related to the disorder studied (in this case, knee OA). Some experts believe that the exact deqi sensations elicited are specific to the physical conditions or the properties of the disorder [6]. It is not surprising that cold sensations were rated the least reliably because they were also rated the least frequently. Sensations such as coldness are generally included in acupuncture sensations scales because coldness and warmth are two of the earliest sensations described in the ancient literature and are symptom specific [2]. For some disorders that present with symptoms such as fever, acupuncture can produce cold sensations to counterbalance these symptoms. The knee OA patients in our study did not tend to report these symptoms during the acupuncture treatment. We speculate that this may be the reason for few patients reporting cold sensations. 

The characterization of deqi sensations is useful for highlighting the differences between real and sham acupuncture experiences. Using a principal component analysis (PCA), we were able to segment the acupuncture sensations into meaningful categories. The results of this study indicate that deqi sensations on the MASS fall into one and/or two categories. One category of sensations we observed includes those sensations related to localized deep pressure. Sensations such as heaviness, fullness/distension, dull pain and cold are common traditional Chinese acupuncture sensations and have previously been reported in relation to deep tissue stimulation [40]. The other category of sensations identified by the PCA includes those sensations related to “spreading sensations” (such as tingling and throbbing). Both of these components are important factors in traditional Chinese acupuncture. The remaining sensations loaded onto both factors and can be viewed as a combination of the two factors. These results are in line with previous studies which have found that certain acupuncture sensations with similar characteristics tend to cluster together [9, 10].

Using the PCA, we were also able to conduct an additional analysis to identify which of the acupuncture sensations might be related to acupuncture mode (real versus sham acupuncture) and to clinical outcomes (KOOS). The present study suggests that patients in the real acupuncture groups report sensations such as soreness and aching significantly more intensely compared to patients in the sham acupuncture group. Exploratory component analyses indicated that tingling and throbbing may be associated with improvements in clinical outcomes.

Researchers continue to debate whether certain sensations or the perceived intensity of those sensations are related to clinical outcomes [17, 20–24]. In this study, we found that real acupuncture, which produced stronger deqi sensations, could also produce significant improvement in pain and function compared with sham acupuncture. This result is consistent with previous studies that indicate that stronger deqi sensation can produce better clinical outcomes [21, 22, 24]. In exploratory analyses, we found that tingling and throbbing were related to clinical outcomes. It is important to note, however, that these analyses are highly exploratory in nature due to small sample size and are not corrected for multiple comparisons. Specific investigation of the relationship between clinical outcomes and deqi sensations is needed to further confirm these findings. 

In an earlier study by Takeda and colleagues, researchers investigated the effect of real and sham acupunctures on osteoarthritis (OA) and found that the experience of deqi can be used as a predictor for significant improvement [17]. In four subsequent OA studies comparing the effect of real acupuncture treatment to sham (minimal depth needling) acupuncture, three studies [21, 22, 41] found that real acupuncture produced significantly better therapeutic effects than sham acupuncture. The fourth study [23] showed no significant difference between real and sham acupuncture treatments and further concluded that “deqi sensation[s] do not result in marked effect,” which calls into questioning “whether deep needling with stimulation and deqi sensation is superior to shallow needling.” 

One potential limitation of this study is the small sample size. We believe, however, that this data will provide the basis for a power analysis of larger clinical trials in the future. 

## 5. Conclusion

In the present longitudinal treatment study, we found that patients with knee OA reliably reported sensations such as heaviness, fullness/distension, aching, and deep pressure that are in coherence with the traditional Chinese acupuncture theory. Compared with sham acupuncture, real acupuncture tends to produce stronger deqi sensation and better clinical outcomes. Soreness and aching were implicated as the two key sensations that differentiate real acupuncture with needle insertion from superficially stimulated acupuncture. Elucidation and characterization of the deqi sensation among patients population may shed new light on our understanding of the mechanism of acupuncture treatment.

## Figures and Tables

**Table 1:** Baseline characteristics.

Mean ± SD	All	High dose	Low dose	Sham
*N *	30	10	10	10
Gender	13 Females	2 Females	7 Females	4 Females
Age	57.5 ± 8.3	60.1 ± 8.9	58.30 ± 8.6	54.1 ± 7.3
Duration (treated knee, years)*	10.5 ± 8.3	9.8 ± 7.4	5.7 ± 6.0	16.22 ± 8.3
KOOS pain	55.94 ± 14.10	58.61 ± 12.99	53.09 ± 9.39	56.11 ± 19.15
KOOS symptoms	52.98 ± 16.17	57.14 ± 19.12	48.21 ± 10.68	53.57 ± 17.82
KOOS ADL	63.58 ± 15.34	66.03 ± 11.83	61.18 ± 13.83	63.53 ± 20.34
KOOS sport^†^	29.48 ± 22.92	30.00 ± 18.11	31.16 ± 19.06	28.50 ± 30.92
KOOS QOL	38.75 ± 15.25	41.88 ± 16.94	38.13 ± 13.96	36.25 ± 15.81

Significant main effect of group (high versus low versus sham) indicated by *; ^†^indicates *N* = 29 due to one missing KOOS subscale score (low-dose group).

**Table 2:** Internal consistency of the 12-item MGH acupuncture sensation scale (MASS).

	Administration 1	Administration 2
Treatment 1	.891	.909
Treatment 2	.948	.963^†^
Treatment 3	.913	.856
Treatment 4	.868	.880^†^
Treatment 5	.893	.907^†^
Treatment 6	.875	.878

All measures of the internal consistency of the MASS administered to 30 subjects are reported as Cronbach's alpha; *N* = 29 due to missing data set indicated by †.

**Table 3:** Test-retest reliability of deqi sensations.

	All	High Dose	Low Dose	Sham
Soreness	.889 (<.001)	.721 (.002)	.925 (<.001)	.688 (.004)
Aching	.913 (<.001)	.726 (.002)	.922 (<.001)	.777 (<.001)
Deep pressure	.903 (<.001)	.808 (<.001)	.943 (<.001)	.821 (<.001)
Heaviness	.928 (<.001)	.88 (<.001)	.926 (<.001)	.769 (<.001)
Fullness/distention	.921 (<.001)	.831 (<.001)	.943 (<.001)	.872 (<.001)
Tingling	.839 (<.001)	.848 (<.001)	.795 (<.001)	.81 (<.001)
Numbness	.861 (<.001)	.839 (<.001)	.837 (<.001)	.932 (<.001)
Sharp pain	.768 (<.001)	.486 (.069)	.812 (<.001)	.761 (.001)
Dull pain	.845 (<.001)	.583 (.026)	.874 (<.001)	.871 (<.001)
Warmth	.74 (<.001)	.664 (.007)	.661 (.008)	.894 (<.001)
Cold	.078 (.37)	.467 (.08)	−.052 (.493)	−.15 (.559)
Throbbing	.792 (<.001)	.774 (<.001)	.601 (.02)	.876 (<.001)
Other	.681 (<.001)	−.75 (.508)	.641 (.011)	.741 (.001)
Sum	.907 (<.001)	.792 (<.001)	.922 (<.001)	.901 (<.001)
Mass index	.907 (<.001)	.764 (.001)	.927 (<.001)	.902 (<.001)

All test-retest reliability analyses reported as intraclass correlation coefficients (*P* value).

**Table 4:** Comparison of intensity ratings and MASS Index across acupuncture treatment groups.

	All	High Dose	Low Dose	Sham
Soreness*	1.29 ± 1.21	1.69 ± .86	1.89 ± 1.47	.28 ± .34
Aching*	1.09 ± 1.14	1.25 ± .8	1.83 ± 1.4	.2 ± .3
Deep pressure	1.05 ± 1.13	1.1 ± .98	1.51 ± 1.53	.55 ± .57
Heaviness	.80 ± 1.12	.67 ± .94	1.54 ± 1.42	.2 ± .25
Fullness/distention	.71 ± 1.04	.68 ± .86	1.25 ± 1.42	.18 ± .38
Tingling	1.21 ± 1	1.61 ± 1.21	1.26 ± 1	.77 ± .64
Numbness	.70 ± .87	.82 ± .89	.96 ± 1.01	.33 ± .61
Sharp pain	1.43 ± 1.05	1.9 ± .84	1.63 ± 1.25	.76 ± .71
Dull pain	1.35 ± 1.1	1.55 ± .78	1.85 ± 1.38	.65 ± .73
Warmth	.48 ± .59	.4 ± .63	.66 ± .54	.38 ± .62
Cold	.06 ± .15	.06 ± .13	.11 ± .22	.02 ± .04
Throbbing*	.63 ± .81	1.13 ± 1.1	.63 ± .53	.15 ± .32
Other	.19 ± .34	.08 ± .19	.12 ± .19	.37 ± .49
Sum score*	10.99 ± 9.3	12.95 ± 7.55	15.22 ± 11.9	4.82 ± 3.79
MASS index*	1.62 ± 1.13	1.96 ± .84	2.10 ± 1.38	.80 ± .62

Values presented as mean ± standard deviation. Significant differences between acupuncture mode (real versus sham acupuncture) after correction for multiple comparisons indicated by ∗.

**Table 5:** By sensation, the number of individuals in each group who reported the sensation at least once across a total of 6 treatment sessions (assessed twice per treatment).

	All	High dose	Low dose	Sham
Soreness*	25	10	9	6
Aching*	21	9	9	5
Deep pressure	27	9	10	8
Heaviness	20	7	7	6
Fullness/distention*	18	8	7	3
Tingling	26	10	8	8
Numbness	20	7	7	6
Sharp pain	28	10	10	8
Dull pain	28	10	10	8
Warmth	17	5	8	4
Cold	8	3	3	2
Throbbing	18	7	7	4
Other	11	2	4	5

Sensations with significantly different frequencies across groups (real versus sham acupuncture) are denoted with a ∗.

**Table 6:** Results of principal component analysis.

Variable	Factor 1	Factor 2
Heaviness	.90	—
Fullness/distension	.87	—
Dull pain	.81	—
Cold	.81	—
Deep pressure	.75	.55
Soreness	.75	.40
Aching	.75	.55
Numbness	.60	.56
Sharp pain	.54	.71
Warmth	.42	.49
Throbbing	—	.95
Tingling	—	.85

**Table 7:** Results of principal component analysis with acupuncture mode (real versus sham) included as an additional variable.

Variables	Factor 1	Factor 2	Factor 3
Heaviness	.90	—	—
Fullness/distension	.88	—	—
Deep pressure	.78	.54	—
Cold	.78	—	—
Dull pain	.77	—	—
Aching	.70	.485	.42
Soreness	.66	—	.56
Numbness	.64	.58	—
Warmth	.52	.60	—
Sharp pain	.50	.64	—
Throbbing	—	.91	—
Tingling	—	.80	—
Acupuncture mode (real versus sham)	—	—	.81

**Table 8:** Results of principal component analysis with the KOOS subscales included as additional variables.

	Pain		Symptom			ADL			Sport			QOL	
	1	2	1	2	3	1	2	3	1	2	3	1	2
KOOS	—	.65	—	—	.96	—	—	−.84	—	—	−.74	—	.76
Soreness	.81	—	.76	—	—	.78	—	—	.80	—	—	.83	—
Aching	.87	—	.76	.53	—	.76	.54	—	.76	.54	—	.86	—
Deep pressure	.88	—	.77	.53	—	.72	.57	—	.70	.55	—	.87	—
Heaviness	.95	—	.91	—	—	.84	—	—	.83	—	—	.93	—
Fullness distension	.93	—	.88	—	—	.81	—	—	.79	—	—	.91	—
Tingling	.47	.72	—	.85	—	—	.82	—	—	.81	—	.46	.73
Numbness	.76	—	.62	.55	—	.54	.57	—	.53	.56	—	.78	—
Sharp pain	.74	.50	.56	.70	—	.59	.68	—	.62	.67	—	.73	.51
Dull pain	.87	—	.82	—	—	.82	—	—	.82	—	—	.86	—
Warmth	.59	—	.45	.47	—	—	.55	.52	—	.54	.69	.56	—
Cold	.73	—	.81	—	—	.87	—	—	.88	—	—	.75	—
Throbbing	—	.84	—	.95	—	—	.94	—	—	.92	—	—	.86
